# Topical Drug Delivery Systems Based on Bacterial Nanocellulose: Accelerated Stability Testing

**DOI:** 10.3390/ijms21041262

**Published:** 2020-02-13

**Authors:** Nuno H. C. S. Silva, Joana P. Mota, Tânia Santos de Almeida, João P. F. Carvalho, Armando J. D. Silvestre, Carla Vilela, Catarina Rosado, Carmen S. R. Freire

**Affiliations:** 1 CICECO–Aveiro Institute of Materials, Department of Chemistry, University of Aveiro, 3810-193 Aveiro, Portugal; nhsilva@ua.pt (N.H.C.S.S.); joao.pedro.carvalho@ua.pt (J.P.F.C.); armsil@ua.pt (A.J.D.S.); cvilela@ua.pt (C.V.); 2 CBIOS–Research Center for Biosciences and Health Technologies, Lusófona University, Campo Grande 376, 1749-024 Lisbon, Portugal; joana.mota@ulusofona.pt (J.P.M.); tania.almeida@ulusofona.pt (T.S.d.A.)

**Keywords:** bacterial nanocellulose, caffeine, lidocaine, ibuprofen, diclofenac, topical drug delivery, storage stability, cutaneous compatibility

## Abstract

Bacterial nanocellulose (BNC) membranes have enormous potential as systems for topical drug delivery due to their intrinsic biocompatibility and three-dimensional nanoporous structure, which can house all kinds of active pharmaceutical ingredients (APIs). Thus, the present study investigated the long-term storage stability of BNC membranes loaded with both hydrophilic and lipophilic APIs, namely, caffeine, lidocaine, ibuprofen and diclofenac. The storage stability was evaluated under accelerated testing conditions at different temperatures and relative humidity (RH), i.e., 75% RH/40 °C, 60% RH/25 °C and 0% RH/40 °C. All systems were quite stable under these storage conditions with no significant structural and morphological changes or variations in the drug release profile. The only difference observed was in the moisture-uptake, which increased with RH due to the hydrophilic nature of BNC. Furthermore, the caffeine-loaded BNC membrane was selected for in vivo cutaneous compatibility studies, where patches were applied in the volar forearm of twenty volunteers for 24 h. The cutaneous responses were assessed by non-invasive measurements and the tests revealed good compatibility for caffeine-loaded BNC membranes. These results highlight the good storage stability of the API-loaded BNC membranes and their cutaneous compatibility, which confirms the real potential of these dermal delivery systems.

## 1. Introduction

Cellulose has been used throughout the years as a substrate for the design of functional blends, composites and hybrid materials in various fields of application [1,2,3]. Additionally, the advent of the nanoscale forms of this ubiquitous natural polymer, namely, cellulose nanofibrils, cellulose nanocrystals and bacterial nanocellulose (BNC), has considerably widened the applications of cellulose [4,5,6,7]. The first two nanocellulose types are typically produced from vegetable cellulose by mechanical, chemical or enzymatic methodologies (or a combination of two or more of these) [8], whereas BNC is biosynthesized by non-pathogenic bacteria such as those belonging to the *Komagataeibacter* genus (formerly known as the *Gluconacetobacter* genus) [9,10].

In the last decade, bacterial exopolysaccharide (viz. BNC) has attracted a great deal of interest in several areas, ranging from nanocomposite materials [11,12,13] to food packaging films [14,15] and conductive membranes for fuel cells [7,16], but, its main application is still in the biomedical field [17,18,19,20]. In fact, there are already BNC-based products being commercialized under the tradename SYNTHECEL^®^ Dura Repair (implant, DePuy Synthes, USA), Epicitehydro (wound dressing, JeNaCell GmbH, Germany) and Celmat (wound dressing, BOWIL, Poland), which are FDA approved and/or CE-certified for biomedical applications [4]. Moreover, BNC is also thriving in the pharmaceutical field for the design of cutaneous drug delivery systems because of its intrinsic and unique features, such as biocompatibility, high water retention capacity and nanostructured porous network [21]. Several studies have confirmed that pure BNC membranes can be successfully loaded with multiple active pharmaceutical ingredients (APIs) (or other bioactive molecules) with different structures, solubility and hydrophilicity. For instance, neat BNC membranes have already been combined with drugs and other bioactive compounds, such as lidocaine [22,23], ibuprofen [23], caffeine [24], diclofenac [25] and amoxicillin [26] in their most common forms or formulated as ionic liquids [27,28] for cutaneous drug delivery. There are also examples of BNC-based nanocomposites being used for the cutaneous delivery of diclofenac [29] and BNC-based hybrid films for the cutaneous delivery of levofloxacin [30].

Despite good in vivo skin compatibility [31] and the demonstrated potential of pure BNC membranes in cutaneous drug delivery of distinct APIs, the stability of these APIs-loaded BNC systems has not yet been fully assessed under diverse storage conditions. In fact, this is a fundamental issue for practical applicability since environmental factors such as ambient temperature, humidity and light might affect the stability of pharmaceutical products, depending on the chemical and physical nature of the API [32]. Therefore, the goal of the present study was to investigate the long-term stability of BNC-based drug delivery systems for three months in the dark under accelerated testing conditions at different relative humidity (RH) and temperature. So, BNC was loaded with distinct model APIs, namely, caffeine, ibuprofen, lidocaine and diclofenac, and the ensuing membranes were characterized in terms of their structure, morphology, moisture-uptake, weight-loss and drug release profile, before and after storage at 75% RH/40 °C, 60% RH/25 °C and 0% RH/40 °C. Additionally, the in vivo cutaneous compatibility of the caffeine-loaded BNC membrane after three months storage in the dark at 75% RH/40 °C was also evaluated.

## 2. Results and Discussion

### 2.1. Preparation and Characterization of API-Loaded BNC Membranes

**Figure 1:**
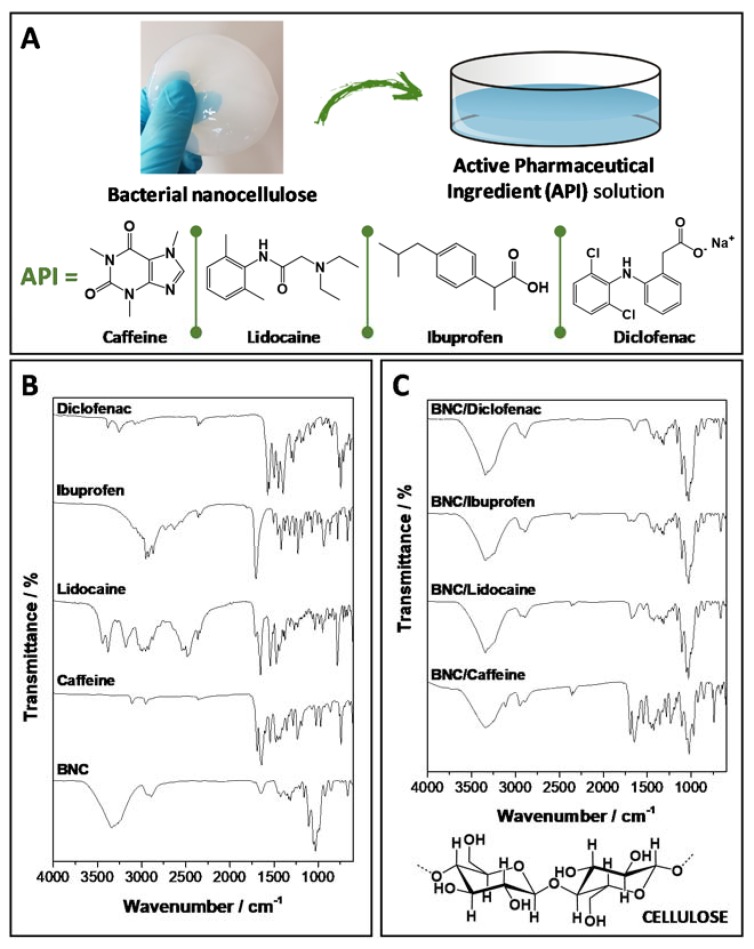
(**A**) Scheme showing the preparation of the active pharmaceutical ingredients (API)-loaded bacterial nanocellulose (BNC) membranes and the chemical structure of all drugs, (**B**,**C**) FTIR-ATR spectra of the individual components, namely, BNC, caffeine, lidocaine, ibuprofen and diclofenac (**B**), and of the four API-loaded BNC membranes with the corresponding chemical structure of cellulose (**C**).

In the present study, BNC membranes were individually loaded with different active pharmaceutical ingredients (APIs), namely, caffeine, lidocaine (in the hydrochloride hydrate form), ibuprofen and diclofenac (in the salt form) (Figure 1A). These four FDA-approved and CE-certified APIs were selected because of their distinct therapeutic effects, solubility and hydrophilicity/hydrophobicity behavior. Caffeine (1,3,7-trimethylxanthine) is the most widely consumed metabolic and central nervous system stimulant drug, it has a well-known effect on lipolytic activity in the adipocytes [33], and is soluble in water (21.7 mg mL^−1^ [34]) and ethanol (ca. 12 mg mL^−1^ [35]); lidocaine (2-diethylamino-*N*-(2,6-dimethylphenyl) acetamide) is a hydrophilic local anesthetic drug with water solubility of 4.1 mg mL^−1^ [34]; ibuprofen (α-methyl-4-(isobutyl)phenylacetic acid) is a hydrophobic non-steroidal anti-inflammatory drug (NSAID) that is highly soluble in ethanol (538 mg mL^−1^ [36]) and poorly soluble in water (21 μg mL^−1^ [34]); and finally, diclofenac (2-[(2,6-dichlorophenyl)amino]benzeneacetic acid) is a NSAID that is insoluble in ethanol and poorly soluble in water in the acidic form (2.37 μg mL^−1^ [34]) but with higher water solubility in the salt form (1.11 mg mL^−1^ [37]).

All four API-loaded BNC membrane systems were prepared via simple diffusion of the corresponding API solution, namely, caffeine, lidocaine and diclofenac in PBS (plus 5% ethanol (w/v) in the case of caffeine) and ibuprofen in absolute ethanol, into the wet BNC network (Figure 1A) according to our previous studies [22,23,24,25]. Glycerol (1% w/v, 2.1 mg per cm^2^ of membrane) was added as a plasticizer to increase the malleability and conformability of the membranes, in view of their cutaneous applications [22,23,24,25], but also as a co-solvent in the case of the BNC/diclofenac membrane, which had a higher glycerol content (5% w/v, 10.4 mg per cm^2^ of membrane). In terms of composition (Table 1), and since the entrapment efficiency was 100% for all API-loaded BNC membranes, the four membrane systems have distinct API doses per area of membrane, i.e., 8.0 mg cm^−2^ for caffeine, 4.2 mg cm^−2^ for lidocaine, 1.9 mg cm^−2^ for ibuprofen and 2.1 mg cm^−2^ for diclofenac. These different compositions were selected based on the equivalent commercial formulations [22,23,24,25].

All API-loaded BNC membranes and their individual components were characterized by Fourier transform infrared-attenuated total reflection (FTIR-ATR) spectroscopy. Figure 1B shows the FTIR-ATR spectra of BNC, caffeine, lidocaine, ibuprofen and diclofenac, whereas Figure 1C displays the FTIR-ATR spectra of the four API-loaded BNC membranes. As anticipated, the infrared spectra of the API-loaded membranes (Figure 1C) exhibit the signature absorption bands of BNC, together with the characteristic vibrations of each API and the intensity of these vibrations are consistent with the different API contents. The cellulosic substrate (Figure 1B) presents absorption bands at 3341 cm^−1^ allocated to the O–H stretching vibration of the primary and secondary hydroxy groups, 2898 cm^−1^ assigned to the stretching vibration of the C–H bonds, 1315 cm^−1^ ascribed to the O–H in the plane bending vibration of the primary and secondary hydroxy groups, 1160 cm^−1^ attributed to the C–O–C antisymmetric stretching vibration of the glycosidic bonds, and 1031 cm^−1^ assigned to the C–O stretching vibration [38]. The absorption bands of glycerol at 3250 cm^−1^ (O–H stretching), 1100 cm^−1^ and 1030 cm^−1^ (C–O stretching vibrations typical of alcohols) [25], are overlapped with the bands of BNC.

On the other hand, the spectra of the APIs exhibit their typical vibrations (Figure 1B) as follows: (i) caffeine: 3108 and 2952 cm^−1^ (C-H stretching from methyl groups), 1690–1428 cm^−1^ (C=N, C=O and C=C bond vibrations) and 1300–1000 cm^−1^ (C-N and C-C bond vibrations); (ii) lidocaine: 3250 cm^−1^ (N-H bonds of secondary amide), 1650 cm^−1^ (C=O bonds of the amide groups) and 1550 cm^−1^ (aromatic C=C bonds); (iii) ibuprofen: 3000 and 2750 cm^−1^ (CH_2_ and CH_3_ stretching), 1705 cm^−1^ (C=O stretching), 1507 cm^−1^ (aromatic C=C bonds), 936 cm^−1^ (CH_3_ rocking vibration) and 668 cm^−1^ (C-H out of plane deformation) [39]; and (iv) diclofenac: 3382 cm^−1^ (N-H stretching), 1603 cm^−1^ (C=C ring skeletal vibration), 1572 cm^−1^ (COO^−^ anti-symmetrical vibration), 1350–1250 cm^−1^ (C-N stretching), and 730–745 cm^−1^ (C-H out-of-plane, di-and tri-substituted rings) [40]. All figures are in agreement with previously published data [22,23,24,25].

**Figure 2:**
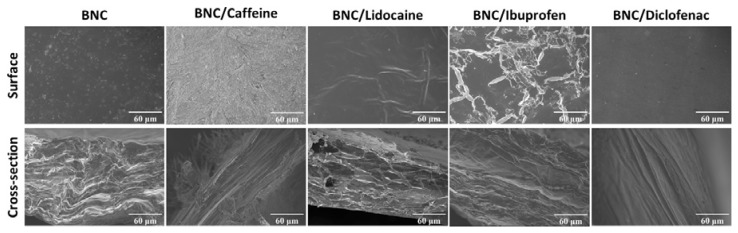
SEM micrographs of the surface and cross-section of the plasticized BNC and API-loaded BNC membranes.

The morphology of the API-loaded BNC membranes was assessed by scanning electron microscopy (SEM) and the surface and cross-sectional micrographs are presented in Figure 2. It is clearly evident that the distinctive morphological features of a pure BNC membrane, namely, the nanofibrillar and lamellar microstructure [4,16], are not observable either on the plasticized BNC membrane or on the four API-loaded BNC membranes. In fact, this is credited to the incorporation of glycerol in the case of the plasticized BNC [31] and the API/glycerol combination in the case of the API-loaded BNC membranes that covered the nanofibrils and filled the lamellar spaces of the exopolysaccharide nanoporous network. This is especially noticeable in the case of the BNC/caffeine membrane composed of the larger amount of API, viz. 8.0 mg of caffeine per cm^2^ of membrane (Table 1). These micrographs are generally in agreement with previously published data [22,23,24,25].

### 2.2. Storage Stability Studies of the API-Loaded BNC Membranes

**Figure 3:**
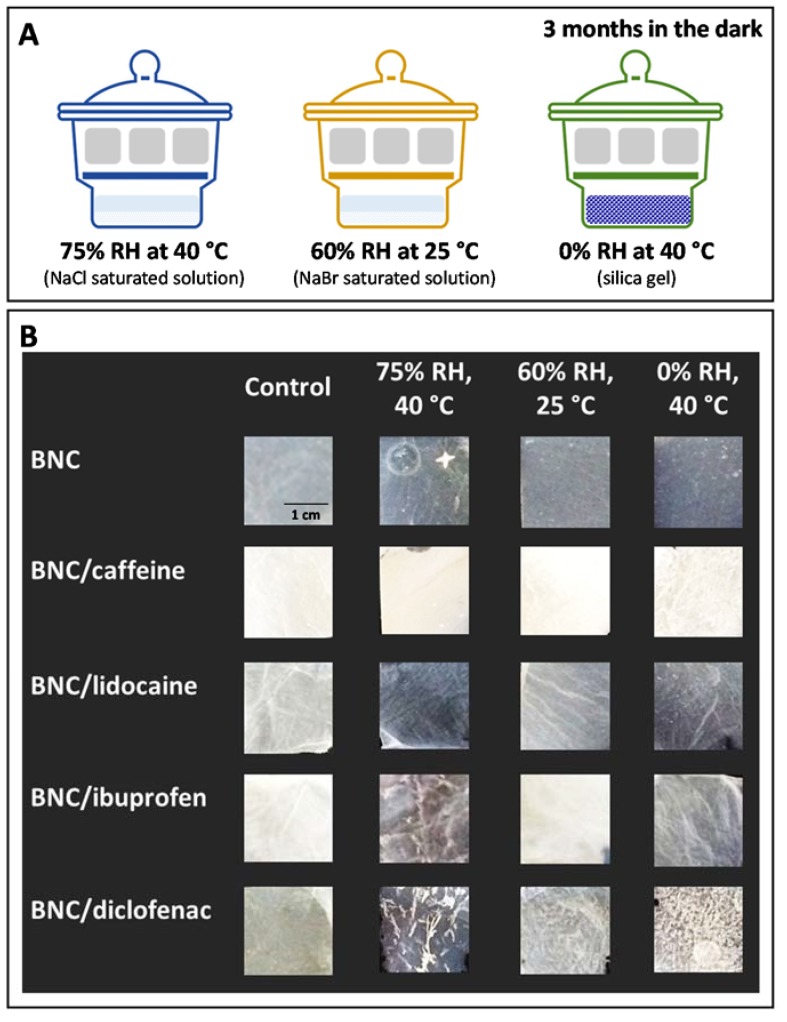
(**A**) Scheme summarizing the conditions for accelerated stability testing (75% RH/40 °C, 60% RH/25 °C, and 0% RH/40 °C), and (**B**) photographs of the API-loaded BNC membranes, before (Control) and after different storage conditions.

According to the WHO Technical Report Series [32], environmental factors, such as ambient temperature, humidity and light, may well alter the stability of pharmaceutical products depending on their chemical and physical properties. Therefore, the API-loaded BNC membranes were exposed to conditions for accelerated stability testing (zone II for temperature and sub-tropical climate zones) [32], namely, at 40 °C/75% RH during three months in the dark, and also at an intermediate condition of 25 °C/60% RH for the same duration of the studies [32] (Figure 3A). An additional test was conducted in the absence of any RH at 40 °C (Figure 3A) to evaluate if a dry atmosphere might be responsible for some weight-loss in the membranes. After subjecting the API-loaded BNC membranes to the above-mentioned conditions, their moisture-uptake, weight-loss, structure, morphology and drug release profile were evaluated in order to check for possible changes.

The simple observation of the API-loaded BNC membranes (Figure 3B) evidence their macroscopic homogeneity, with the exception of the BNC/diclofenac membrane where some white diclofenac clusters were visible in the membranes after being exposed to different environmental humidity and temperature. This behavior might be attributed to crystallization or precipitation of the API, which increases with humidity reduction. Moreover, the API-loaded BNC membranes stored for three months at 0% RH/40 °C appear to be more brittle than those stored at 60% RH/25 °C and 75% RH/40 °C, probably because the moisture-uptake promoted a plasticizing effect on the membranes, as described for other BNC-based membranes [41,42].

Since these API-loaded BNC membranes were submitted to different levels of environmental humidity, it is relevant to measure their moisture-uptake after three months under different storage conditions. Overall, the moisture-uptake increased with increasing RH (from 60% to 75%) for all membrane samples (Table 2). While BNC plasticized with 1% (w/v) of glycerol (a well-known humectant [43]) reached moisture-uptake values of 9.9 ± 3.1% at 60% RH/25 °C and 22.3 ± 0.4% at 75% RH/40 °C, the BNC containing 5% (w/v) glycerol reached a similar value at 60% RH/25 °C (10.3 ± 1.4%) but a higher moisture-uptake at 75% RH/40 °C (35.7 ± 1.9%). These values are analogous to those recently reported for a pure BNC membrane (without glycerol) at 98% RH for 48 h [15,16].

Regarding the API-loaded BNC membranes, the moisture-uptake is dependent on the API nature with lidocaine and diclofenac exhibiting the highest moisture-uptake values particularly at higher RH and temperature, namely, 19.8 ± 4.1% at 60% RH/25 °C and 36.3 ± 2.1% at 75% RH/40 °C for BNC/lidocaine, and 9.2 ± 1.0% at 60% RH/25 °C and 31.3 ± 7.3% at 75% RH/40 °C for BNC/diclofenac (Table 2). These data are clearly related with the fact that the former is in the hydrochloride hydrate form, while the latter is in the sodium salt form, which on the whole leads to higher hygroscopicity of the former, as discussed elsewhere [44]. On the other hand, the caffeine-loaded BNC membrane presents a lower moisture-uptake with values of 16.5 ± 0.7% at 60% RH/25 °C and 26.0 ± 4.5% at 75% RH/40 °C, whereas the ibuprofen-loaded BNC membrane exhibits the lowest moisture-uptake values, i.e., 4.1 ± 1.4% at 60% RH/25 °C and 12.3 ± 2.9% at 75% RH/40 °C (Table 2), due to the hydrophobic character of the drug. It should be noted that the diclofenac-loaded BNC membrane is the less affected by the storage conditions, as the moisture-uptake values (9.2 ± 1.0% at 60% RH/25 °C and 31.3 ± 7.3% at 75% RH/40 °C, Table 2) are not significantly different from those of the BNC membrane plasticized with 5% (w/v) glycerol without the API (BNC5: 10.3 ± 1.4% at 60% RH/25 °C and 35.7 ± 1.9% at 75% RH/40 °C, Table 2).

When the storage conditions were changed to 0% RH at 40 °C for three months, all samples exhibited weight-loss, most likely due to the removal of adsorbed water and glycerol. It is evident that the incorporation of the APIs into the plasticized BNC membranes reduced their weight-loss values from 27.0 ± 8.0% for the BNC plasticized with 1% (w/v) of glycerol to a minimum of 5.2 ± 0.6% for BNC/ibuprofen, and also from 37.2 ± 10.1% for the BNC containing 5% (w/v) glycerol to 25.4 ± 0.7% for BNC/diclofenac (Table 2).

**Figure 4:**
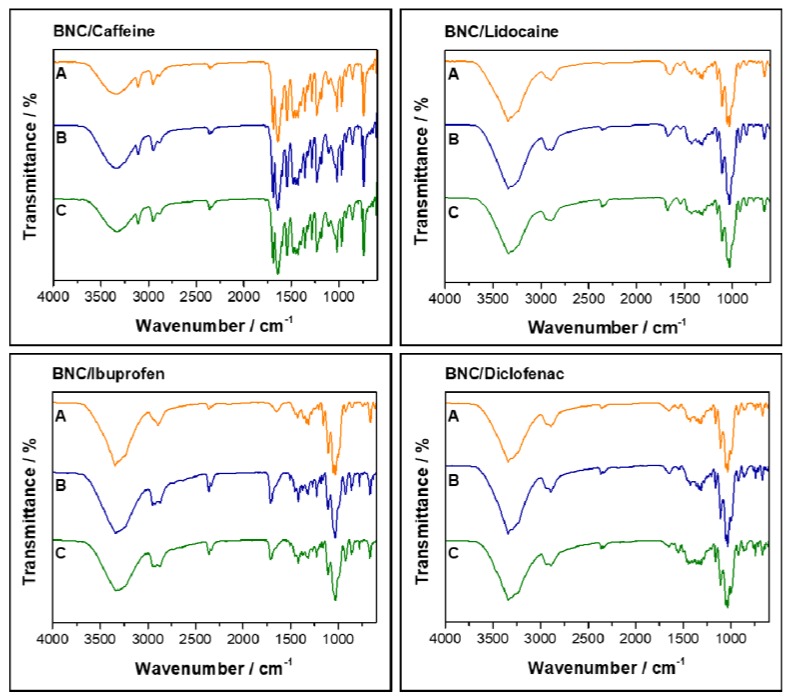
FTIR-ATR spectra of the API-loaded BNC membranes stored for three months in different conditions: (A) 75% RH at 40 °C, (B) 60% RH at 25 °C and (C) 0% RH at 40 °C.

In order to investigate possible interactions and/or degradation phenomena occurring after exposing the API-loaded membranes to distinct storage conditions, their structure was once again studied by FTIR-ATR (Figure 4). The spectra of the API-loaded membranes after storage are very similar to those of the starting materials (Figure 1C), independently of the storage conditions, which suggests that the membranes and/or their precursors do not suffer any structural change under the studied conditions. Furthermore, and despite the moisture-uptake capacity (Table 2) of these API-loaded membranes, there are no peak shifts or differences in the intensity of the absorption bands, which points to stable membranes under the studied storage conditions, at least from a structural point-of-view.

**Figure 5:**
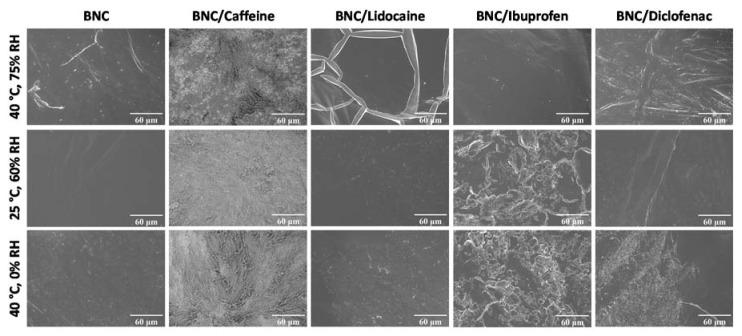
Surface SEM micrographs for BNC and API-loaded BNC membranes after 3 months storage in different conditions.

The morphology of the API-loaded BNC membranes was again verified by SEM after storing the membranes at 75% RH/40 °C, 60% RH/25 °C and 0% RH/40 °C for three months in the dark, as shown in Figure 5. Overall, the comparison of the surface micrographs before (Figure 2) and after (Figure 5) storage at different RH and temperature conditions demonstrates that there are no relevant differences, although the surface of some of the membranes at higher RH seem more homogenous. Moreover, the micrographs show good dispersion of each API in the BNC membranes, which is in accordance with the non-existence of structural changes assessed by infrared vibrational spectroscopy (Figure 4).

**Figure 6:**
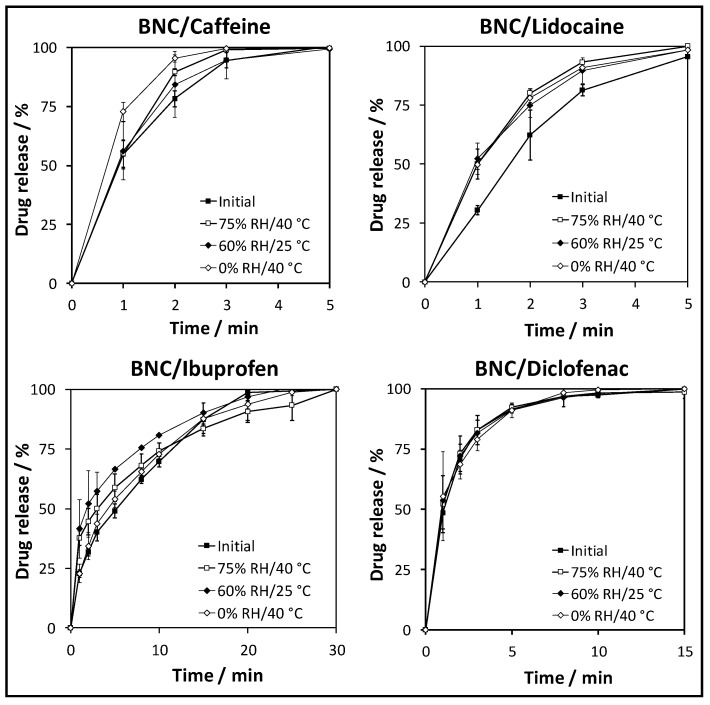
Drug release profiles of the API-loaded BNC membranes at time 0 (initial) and after 3 months under conditions for accelerated stability testing, namely, 75% RH/40 °C, 60% RH/25 °C and 0% RH/40 °C; the values are the mean of three replicates and error bars represent the standard deviations.

Despite the absence of visible changes at the structural and morphological level, it is necessary to confirm that the in vitro release profiles of the API-loaded BNC membranes remain the same after three months under different storage conditions. These assays were carried out in PBS at pH 7.4 to simulate the pH of the skin, before and after storing the membranes at 75% RH/40 °C, 60% RH/25 °C and 0% RH/40 °C, as summarized in Figure 6. Generally, the initial drug release curves of the four membrane systems show a typical release profile with a burst, followed by a plateau where the drug release rate reaches a maximum value.

Furthermore, all membranes achieved a total release (100%) of the respective API after 5 min in the case of caffeine and lidocaine, 30 min for ibuprofen and 15 min for diclofenac (Figure 6). These differences in release time demonstrate the effect of the solubility of each API in the large release media volumes used in these assays. In fact, caffeine (21.7 mg mL^−1^ [34]) and lidocaine (4.1 mg mL^−1^ [34]) are the two APIs with high water solubility, followed by diclofenac with lower water solubility (salt form: 1.11 mg mL^−1^ [37]) and ibuprofen, which has poor water solubility (21 μg mL^−1^ [34]). Therefore, the release of the four APIs from the BNC membranes is basically governed by diffusion through the porous and three-dimensional BNC network, which is favored by the high swelling capacity of BNC [22,24,25]. These initial drug release profiles of the API-loaded BNC membranes are analogous to those previously published for API-loaded BNC membranes composed of caffeine [24], lidocaine [22] and diclofenac [25].

In addition, the initial drug release profiles of the API-loaded BNC membranes can be fitted to the Korsmeyer–Peppas kinetic model [45,46] according to the following equation: $M_{t}/M_{\infty }=kt^{n}$, where $M_{t}$ is the amount of API released at time t, $M_{\infty }$ is the amount of API released at infinite time, k is the kinetic constant and n is the diffusion constant indicating the release mechanism [45,47]. According to this model, only $M_{t}/M_{\infty }<60\%$ has to be contemplated for fitting, and therefore, a release exponent (*n*) of 0.52 (regression coefficient: *R*^2^ = 0.999) was achieved for BNC/caffeine, 0.91 (*R*^2^ = 0.988) for BNC/lidocaine, 0.48 (*R*^2^ = 0.998) for BNC/ibuprofen and 0.55 (*R*^2^ = 0.999) for BNC/diclofenac. These fitting parameters are indicative of Fickian diffusion (i.e., diffusion-controlled drug release) in the case of BNC/ibuprofen, and non-Fickian transport (0.5 < *n* < 1.0, i.e., diffusion- and swelling-controlled drug release) in the case of BNC/caffeine, BNC/lidocaine and BNC/diclofenac [45,46,47].

A look at the drug release profiles after exposure to the accelerated testing conditions shows that the BNC/diclofenac membrane experienced the smallest effects with almost no difference in the amount of API liberated as a function of time. The other three API-loaded BNC membrane systems experienced some changes that resulted in faster release rates. For example, after storing the membranes at 75% RH/40 °C for three months in the dark, the release of (i) caffeine increased from 78 ± 3% to 90 ± 1% after 2 min, (ii) lidocaine increased from 62 ± 10% to 80 ± 2% after 2 min, and (iii) ibuprofen increased from 32 ± 1% to 45 ± 7% after 2 min. These differences can probably be attributed to a higher API content at the surface of the membranes due to migration during storage followed by precipitation at the surface. Lastly, the fitting parameters to the Korsmeyer—Peppas kinetic model [45,46] were also maintained in the range of the Fickian diffusion for BNC/ibuprofen and non-Fickian transport in the case of BNC/caffeine, BNC/lidocaine and BNC/diclofenac. Overall, the four API-loaded BNC membranes that were studied in the present work are stable under the accelerated stability testing conditions, namely, at 75% RH/40 °C, 60% RH/25 °C and 0% RH/40 °C for three months in the dark, with no relevant changes in structure, morphology and in vitro drug release profile.

It is worth noting that the rapid release profile that was observed is suitable for the therapeutic effect intended for the APIs used in this study. In order to achieve a rapid local anesthetic, analgesic and anti-inflammatory action, a fast-transcutaneous penetration must be ensured, which is only possible if a sufficient amount of drug is released from the delivery system. This scenario is also applicable in the case of caffeine, where the API has to reach the innermost layer of the skin, the adipose tissue.

### 2.3. In Vivo Assessment of Cutaneous Compatibility

**Figure 7:**
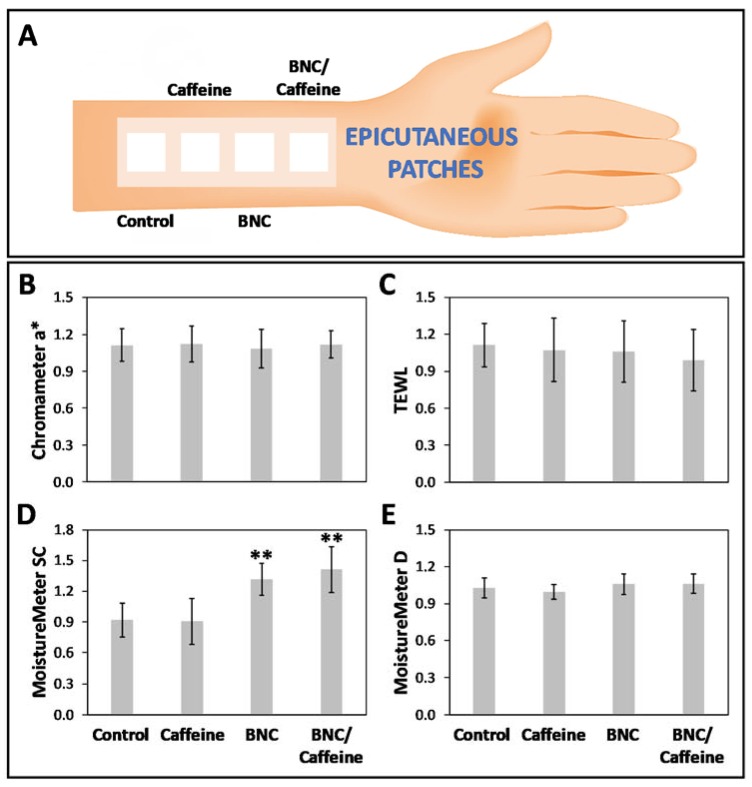
Cutaneous compatibility data obtained with 20 human volunteers (male and female): (**A**) erythema (skin irritation), (**B**) skin barrier function, (**C**) stratum corneum hydration, and (**D**) deep skin hydration; all values are the means and the error bars represent the standard deviation; the double asterisk (**) denotes statistically significant differences to the negative control (i.e., distilled water) (*p* < 0.05).

BNC is known for exhibiting in vitro compatibility with epidermal cells (e.g., human HaCaT keratinocyte cell line [28,29]), but most importantly, it has good in vivo compatibility [31]. So, the in vivo cutaneous compatibility of the BNC-based membranes was tested by applying epicutaneous patches in the volar forearm (Figure 7A) of twenty human volunteers (male and female, mean age: 28.8 ± 8.6 years) for 24 h. Visual scoring and non-invasive biophysical measurements were conducted at each application site in order to establish any impact on the skin [48], namely, stratum corneum (SC) and deep skin hydration, the skin barrier function in the form of transepidermal water loss (TEWL) and erythema (a*) as the indicator of skin redness [49,50,51]. All measurements were conducted 2 h after patch removal in order to eliminate possible interference from the skin stripping that the patch removal inevitably causes. The effect of inter-individual variability was reduced by analyzing the results as the ratio between the values obtained after patch application and the basal values, as portrayed in Figure 7B–E. Herein, the in vivo tests were only carried out for the pure BNC membrane plasticized with 1% glycerol and caffeine-loaded BNC membrane after three months storage in the dark at 75% RH/40 °C. A control sample (i.e., distilled water) and an aqueous solution of caffeine (2% w/v) were used as negative control and for comparison purposes, respectively. The reason for selecting the caffeine-loaded BNC membrane for this study is associated with the fact that caffeine is largely consumed worldwide in several beverages (e.g., coffee, tea and energizing drinks) and has been widely investigated for dermal and cosmetic applications [33], therefore, it has a more favorable toxicity profile. The other APIs, namely, the NSAIDs ibuprofen and diclofenac, have known side-effects on the skin [52,53,54], whereas the anesthetic effect of lidocaine could mask some unexpected reactions, for instance, itching or pain. Therefore, the caffeine-loaded BNC membrane was used as a proof-of-concept to demonstrate that the absence of any impact on the structure, morphology and drug release profile after accelerated stability testing also translates into membranes with good in vivo cutaneous compatibility.

In the visual assessment, both plasticized BNC and caffeine-loaded BNC membranes performed well in the compatibility tests with all volunteers scoring zero cutaneous responses at every test site according to the International Contact Dermatitis Research Group (ICDRG) scale [55]. Starting with the control (i.e., distilled water) and the caffeine solution, neither have any impact on the skin, namely, SC and deep skin hydration, the barrier function and erythema, which was expected given that the former is water and the latter is already used in commercial aqueous and gel formulations in products for cellulite management [24].

Regarding the plasticized BNC membrane, it was very clear that this exopolysaccharide did not promote any skin irritation (a*, Figure 7B), compromise the skin barrier (TEWL, Figure 7C) or affect the deep skin hydration (Figure 7E). Nevertheless, the presence of glycerol, which is a recognized moisturizer in skin care preparations [56], increased the SC hydration (Figure 7D) from 0.92 ± 0.17 for the control to 1.32 ± 0.15 for BNC. These data are in accordance with the in vivo study in humans conducted by Almeida et al. [31] where the skin compatibility of pure BNC membranes plasticized with glycerol (1% w/v) showed skin moisturization properties, and no damaging effects were detected for any of the other cutaneous properties tested, namely, TEWL and erythema [31].

As in the case of the plasticized BNC membrane, the non-invasive measurements for the caffeine-loaded BNC membrane established that it did not cause any alterations of the skin at any of the tested sites. The basal values of skin redness (a*) were preserved after membrane application, which indicates that it did not cause any erythema, viz. no skin irritation (Figure 7B). Furthermore, the skin barrier function was well-preserved, since the TEWL was unaffected (Figure 7C) and there were no apparent changes in the water content of the deeper skin layers, which is also proof that no irritation or inflammation was triggered, even at a deeper level of the skin (Figure 7E). Nevertheless, a significant impact on the superficial hydration was observed at the sites where the membrane was applied, with an increase in the moisture content of the SC (Figure 7D, *p* < 0.05) from 0.92 ± 0.17 for the control to 1.41 ± 0.22 for BNC/caffeine. This effect is also attributable to the moisturizing effect of glycerol, which has a dual effect in topical delivery systems, namely, plasticizer and moisturizer.

## 3. Materials and Methods

### 3.1. Chemicals

Citric acid (≥99.5%), glucose (≥99.5%), potassium sulphate (≥99.0%), sodium phosphate dibasic (≥99.0%), caffeine (99%), ibuprofen (≥98%), lidocaine hydrochloride monohydrate (99.5%), diclofenac sodium salt (≥98.5%) and glycerol (≥99.5%) were purchased from Sigma-Aldrich (Sintra, Portugal). Bacteriological agar, peptone and yeast extract were acquired from Himedia Laboratories GmbH (Einhausen, Germany). Phosphate buffer saline (PBS, pH 7.4) was supplied from Gibco^®^ (Life Technologies, Carlsbad, CA, USA). Ultrapure water (Type 1, 18.2 MΩ·cm resistivity (25 °C) at 0.5 L min^−1^) was purified by a Simplicity^®^ Water Purification System (Merck, Darmstadt, Germany). All other chemicals and solvents were of laboratory grade.

### 3.2. Bacterial Nanocellulose Biosynthesis

Wet membranes of bacterial nanocellulose (BNC) characterized by a three-dimensional network of nano- and microfibrils, were biotechnologically produced in our laboratory using the *Gluconacetobacter sacchari* bacterial strain [57]. Briefly, the bacteria were incubated in a Hestrin-Schramm (HS) liquid medium (20 g L^−1^ glucose, 5 g L^−1^ peptone, 5 g L^−1^ yeast extract, 2.7 g L^−1^ Na_2_HPO_4_, 1.15 g L^−1^ citric acid, and 15 g L^−1^ agar, pH 5) under static conditions. After incubation for several days at 30 °C, the BNC membranes were separated from the media, treated with 0.5 M NaOH aqueous solution and repeatedly washed with ultrapure water to remove culture media components. Lastly, the membranes were whitened with 1% sodium hypochlorite solution, washed several times with water until neutral pH, and stored in ultrapure water in the refrigerator until further use.

### 3.3. Preparation of API-loaded BNC Membranes

BNC membrane discs (diameter: ca. 7 cm) were individually loaded with different APIs, namely, caffeine, lidocaine, ibuprofen and diclofenac, as described in other studies [22,23,24,25]. The list of the prepared membranes with the respective API dose is summarized in Table 1. In brief, drained BNC membranes (with a water content of only 40%) were soaked in 8 mL of PBS solution containing the corresponding API and glycerol as plasticizer. For one of the pure wet BNC membranes, the water content was replaced by ethanol before the inclusion of ibuprofen. Given the different water solubilities of the four APIs, the following four solutions were prepared: (i) 3.85% caffeine (w/v) in PBS solution containing 5% ethanol (w/v) and 1% glycerol (w/v), (ii) 2% lidocaine (w/v) in PBS solution with 1% glycerol (w/v), (iii) 1% ibuprofen (w/v) in ethanol with 1% glycerol (w/v), and (iv) 1% diclofenac solution (w/v) in PBS with 5% glycerol (w/v). After the complete absorption of the corresponding solution (viz. 100% entrapment efficiency), the API-loaded BNC membranes were placed in a Petri dish and dried at 50 °C in a ventilated oven (Thermo Fisher Scientific, Waltham, MA, USA) for 24 h. The dried API-loaded BNC membranes were stored in a desiccator until further use. For comparison purposes, two BNC membranes containing 1% and 5% glycerol (w/v) without any API were also prepared.

The entrapment efficiency of each API-loaded BNC membrane was calculated as follows:

(1) $$\mathrm{Entrapment}\;\mathrm{efficiency}\;\left(\%\right)=\frac{W_{\mathrm{loaded}}}{W_{\mathrm{initial}}}\times 100$$

where $W_{\mathrm{loaded}}$ is the API loaded weight and $W_{\mathrm{initial}}$ is the API initial weight.

The drug loading of each API-loaded BNC membrane was determined in terms of API mass per area of BNC membrane, according to the following equation:

(2) $$\mathrm{Drug}\;\mathrm{loading}\;\left(\mathrm{mg}\;{\mathrm{cm}}^{-2}\right)=\frac{W_{\mathrm{loaded}}}{\mathrm{area}\;\mathrm{of}\;\mathrm{BNC}\;\mathrm{membrane}}$$

### 3.4. Characterization Techniques

Fourier transform infrared-attenuated total reflection (FTIR-ATR) spectra were collected in the range of 600–4000 cm^−1^ at a resolution of 4 cm^−1^ over 32 scans in a Perkin-Elmer FT-IR System Spectrum BX spectrophotometer (Perkin-Elmer, Waltham, MA, USA) equipped with a single horizontal Golden Gate ATR cell.

Surface and cross-section micrographs of the membranes were obtained by an ultra-high-resolution field-emission SEM Hitachi SU-70 microscope (Hitachi High-Technologies Corporation, Tokyo, Japan) operating at 4 kV. The membranes for surface and cross-section (fractured in liquid nitrogen) examination were placed on a steel plate and coated with a carbon film prior to analysis.

### 3.5. Storage Stability Tests

Samples (1 × 1 cm^2^) of each API-loaded BNC membrane were placed in glass vials and stored for 3 months in the dark (to avoid the effect of light, as requested in the storage of many drugs) at different relative humidity (RH) and temperature conditions. So, the membranes were tested at 0% RH (using a desiccator with silica gel) and 40 °C, 60% RH (using a sodium bromide saturated solution [58]) and 25 °C, and 75% RH (using a sodium chloride saturated solution [58]) and 40 °C. Relative humidity was checked periodically with a humidity meter (VWR^®^ Traceable^®^ Hygrometer, VWR International, Alfragide, Portugal) to ensure constant humidity throughout the study. The assay was conducted in triplicate. All samples were weighed before and after storage. The moisture-uptake of the membrane samples stored at 75% and 60% RH was calculated by the equation:

(3) $$\text{Moisture-uptake}\;\left(\%\right)=\frac{W_{\mathrm{stored}}-W_{\mathrm{initial}}}{W_{\mathrm{initial}}}\times 100$$

where $W_{\mathrm{initial}}$ is the initial weight of the dry membrane and $W_{\mathrm{stored}}$ is the weight after three months of storage under specific conditions. The weight-loss of the membranes stored at 0% RH were calculated as follows:

(4) $$\text{Weight-loss}\;\left(\%\right)=\frac{W_{\mathrm{initial}}-W_{\mathrm{stored}}}{W_{\mathrm{initial}}}\times 100$$

### 3.6. In Vitro Drug Release Assays

Before the drug release studies, the stored membranes were equilibrated in a desiccator filled with silica gel for 24 h. Drug release studies were performed in 200 mL of PBS, pH 7.4 at 32 °C [59], and the receptor medium was stirred at 130 rpm. At pre-determined time intervals, samples of 2 mL were collected and replaced with fresh and pre-heated medium. Quantitative analysis of the drugs was performed by ultraviolet-visible spectroscopy (Thermo Scientific Evolution 600, Thermo Fisher Scientific, Waltham, MA, USA), at 273 nm for caffeine [24], 230 nm for lidocaine [22,23], 225 nm for ibuprofen [23], and 276 nm for diclofenac [25]. Six replicates were performed for each membrane.

### 3.7. In Vivo Assessment of Cutaneous Compatibility of Caffeine-Loaded BNC Membranes

Twenty human volunteers (male and female, mean age: 28.8 ± 8.6 years) participated in this study, after informed oral and written consent. The study was approved by the local Ethics Committee of the Faculty of Health Sciences of the Lusófona University (Lisbon, Portugal) and was performed in accordance with the ethical standards as laid down in the 1964 Declaration of Helsinki and its later amendments [60].

Epicutaneous patches (1 × 1 cm^2^, Finn Chambers^®^, Epitest Ltd Oy, Tuusula, Finland) were applied in the volar forearm of the volunteers for 24 h. Each patch had 4 chambers containing a caffeine-loaded BNC membrane, a pure BNC membrane, distilled water (negative control) and a 2% (w/v) caffeine aqueous solution. A visual assessment of each application site was conducted 2 h after the patch removal and cutaneous responses were classified according to the International Contact Dermatitis Research Group criteria [55]. Additionally, non-invasive measurements were conducted in each application site in order to further establish any impact on the skin [48]. Stratum corneum and deep skin hydration were measured with a Moisture Meter-SC and a Moisture Meter-D (Delfin Technologies, Kuopio, Finland), respectively. Barrier function, in the form of transepidermal water loss (TEWL), was assessed with a Tewameter TM300 (CK Electronics, Koln, Germany) and erythema, the redness of the skin, was probed with a Chromameter CR300 (Minolta, Osaka, Japan), by quantifying the a* values. Measurements were performed before patch application and 2 h after patch removal, following published guidelines [49,50,51]. To minimize the effect of inter-individual variability, the results were analyzed as the ratio between the values obtained after patch application and the basal values.

### 3.8. Statistical Analysis

Analysis of variance (ANOVA) and Tukey’s test (OriginPro, version 9.0.0, OriginLab Corporation, Northampton, MA, USA) was used to determine the statistical significance established at *p* < 0.05.

## 4. Conclusions

The long-term storage stability of BNC membranes loaded with different APIs, namely, caffeine, lidocaine, ibuprofen and diclofenac was investigated. The storage stability was assessed under accelerated testing conditions at different temperatures and relative humidity (RH). All systems were stable under the studied storage conditions with no substantial structural and morphological changes or variations in the drug release profile. The only change was in the moisture-uptake, which increased with RH due to the hydrophilic nature of BNC. Moreover, the caffeine-loaded BNC membrane was chosen for in vivo cutaneous compatibility studies, where patches were applied in the volar forearm of twenty volunteers for 24 h. The cutaneous responses were measured by non-invasive methodologies and the in vivo tests showed good compatibility for caffeine-loaded BNC membranes. These results highlight the good storage stability of the APIs-loaded BNC membranes and validate the actual potential of these topical delivery systems with different APIs.

## Figures and Tables

**Table 1:** Identification of the API-loaded BNC membranes with the respective drug dose.

Membrane	Glycerol ^1^		Drug Dose ^2^	
	% (w/v)	mg cm^−2^	% (w/v)	mg cm^−2^
BNC1 ^3^	1.0	2.1	–	–
BNC/Caffeine	1.0	2.1	3.8	8.0
BNC/Lidocaine	1.0	2.1	2.0	4.2
BNC/Ibuprofen	1.0	2.1	0.9	1.9
BNC5 ^3^	5.0	10.4	–	–
BNC/Diclofenac	5.0	10.4	1.0	2.1

^1^ Glycerol: % on a w/v basis relative to the volume of the solution, and mg cm^−2^ relative to the surface area of the membrane; ^2^ drug dose: % on a w/v basis relative to the solution volume, and mg cm^−2^ relative to the surface area of the membrane; ^3^ BNC1 and BNC5 are the pure BNC membranes plasticized with 1% and 5% (w/v) of glycerol, respectively.

**Table 2:** Water-uptake and weight-loss after three months storage for plasticized BNC and API-loaded BNC membranes.

Membrane ^1^	Moisture-Uptake/% ^2^		Weight-Loss/% ^2^
	75% RH at 40 °C	60% RH at 25 °C	0%RH at 40 °C
BNC1	22.3 ± 0.4	9.9 ± 3.1	27.0 ± 8.0
BNC/Caffeine	26.0 ± 4.5	16.5 ± 0.7 *	11.8 ± 5.0 *
BNC/Lidocaine	36.3 ± 2.1 *	19.8 ± 4.1 *	7.8 ± 3.0 *
BNC/Ibuprofen	12.3 ± 2.9 *	4.1 ± 1.4 *	5.2 ± 0.6 *
BNC5	35.7 ± 1.9	10.3 ± 1.4	37.2 ± 10.1
BNC/Diclofenac	31.3 ± 7.3	9.2 ± 1.0	25.4 ± 0.7 *

^1^ See Table 1 for membrane identification; ^2^ the values are the means of triplicates with the respective standard deviations; the asterisk (*) denotes statistically significant differences to the plasticized BNC membrane (1% or 2% w/v glycerol) (*p* < 0.05).

## Abbreviations

APIs	Active pharmaceutical ingredients
BNC	Bacterial nanocellulose
CE	Conformité Européenne
FDA	Food and Drug Agency
FTIR-ATR	Fourier transform infrared-attenuated total reflectance
ICDRG	International Contact Dermatitis Research Group
NSAID	Non-steroidal anti-inflammatory drug
PBS	Phosphate buffer saline
RH	Relative humidity
SC	Stratum corneum
SEM	Scanning electron microscopy
TEWL	Transepidermal water loss
WHO	World Health Organization
